# Preoperative antibiotic prophylaxis regimen in brain tumour surgery in Sweden: a quasi-experimental study

**DOI:** 10.1007/s00701-020-04309-6

**Published:** 2020-05-07

**Authors:** Simon Skyrman, Jiri Bartek, Maryam Haghighi, Ida Fornebo, Tomas Skoglund, Asgeir Store Jakola, Ann-Christin von Vogelsang, Petter Förander

**Affiliations:** 1 Department of Neurosurgery, Karolinska University Hospital, 171 76 Stockholm, Sweden; 2 Department of Clinical Neuroscience, Karolinska Institutet, Stockholm, Sweden; 3 Department of Neurosurgery, Rigshospitalet, Copenhagen, Denmark; 4 Department of Clinical Neuroscience and Department of Medicine, Karolinska Institutet, Stockholm, Sweden; 5 Department of Neurosurgery, Sahlgrenska University Hospital, Gothenburg, Sweden; 6 Institute of Neuroscience and Physiology, Department of Clinical Neuroscience, Sahlgrenska Academy, University of Gothenburg, Box 430, 40530 Gothenburg, Sweden

**Keywords:** Antibiotic prophylaxis, Neurosurgery, Craniotomy, Surgical site infection, Cefuroxime, Cloxacillin,

## Abstract

**Background:**

There has been varied clinical practice concerning antibiotic prophylaxis in patients undergoing craniotomy. In Sweden, both Cloxacillin and Cefuroxime have frequently been used. We aimed to study the clinical effectiveness of these two regimens.

**Methods:**

A quasi-experimental design was used. The sample consisted of 580 adult (> 18 years) patients operated 2012–2015, of which 375 received Cloxacillin (pre-intervention group) and 205 received Cefuroxime (intervention group). Primary endpoint was the incidence of surgical site infection (SSI) 12 months after surgery, while secondary endpoints were the need for reoperation due to SSI, the amount antibiotics used and the number of visits in the outpatient clinic related to SSI. A control group from another institution was reviewed to rule out clinical trial effects.

**Results:**

When analysed by intention to treat, the pre-intervention group had a significant higher incidence of SSI, 13.3% (50/375) vs 5.4% (11/205) in the intervention group (*p* < 0.01). A treatment per protocol analysis confirmed the result. The number of reoperations due to SSI were significantly reduced in the intervention group, 3.4% (7/205) vs 8.3% (31/375) (*p* = 0.02), as was the total antibiotic use (*p* = 0.03) and the number of visits in the outpatient clinic (*p* < 0.01). In the control group, the reoperation rate as result of SSI was lower (*p* = 0.02) prior to the opposite change from Cefuroxime to Cloxacillin, 1.8% (27/1529) vs 3.1% (43/1378).

**Conclusion:**

In Sweden, Cefuroxime as prophylaxis in brain tumour surgery by craniotomy seems to be superior to Cloxacillin.

## Introduction

Surgical site infection (SSI) is a serious threat to neurosurgical patients causing increased morbidity and mortality [1, 23], with a reported risk ranging from 1 to 13% [1, 5, 25, 30]. SSI is a common complication after neurosurgical interventions, causing a significant economic impact on the health care system [28], calculated to be on average > 10,000 EURO in case of SSI after a craniotomy [25]. SSIs after craniotomy may require multiple surgical interventions, such as surgical wound debridement, bone flap removal and cranioplasty [12, 35] with risk of affecting the post-operative cosmetic result [16]. Further, for patients with intracranial tumours, SSI may postpone critical adjuvant oncologic treatment, as radiation- and chemotherapy must be withheld until infection resolution [35].

The Center for Disease Control’s (CDC) guidelines provide recommendations of how to define, prevent and report SSI [7, 21]. A central part of SSI prevention is the administration of an antimicrobial agent preoperatively. There is robust evidence supporting the use of prophylactic antibiotic in neurosurgical procedures [4, 5, 7, 11, 20], and the effectiveness of several different systemic [8, 18, 20, 33] as well as topical antimicrobial agents [10, 22] has been studied. In Scandinavia, the most common prophylactic antibiotic regimen in neurosurgery consists of intravenous Cloxacillin or Cefuroxime; however, no specific antibiotic regimen has proven better than the other [4, 5].

The aim of this study was to evaluate the effect of a change in antibiotic prophylaxis regimen from Cloxacillin to Cefuroxime in patients with intracranial tumours, treated surgically with a craniotomy.

## Material and methods

### Study design

The study had a retrospective, quasi-experimental design, where the intervention consisted of a change in the antibiotic prophylaxis regimen at the Department of Neurosurgery at the Karolinska University Hospital (Stockholm, Sweden). Until April 2014, the standard prophylaxis regimen was 2 g intravenous infusion of Cloxacillin (Isoxazolyl penicillin) 1 h before the start of surgery. From May 2014 and onwards, patients instead received 1.5 g intravenous infusion of Cefuroxime (2nd generation cephalosporin) 1 h before the start of surgery. Thus, patients in the *pre-intervention group* were scheduled for routine Cloxacillin while patients in the *intervention* group received routine Cefuroxime. For patients not tolerating the standard drugs, 600 mg iv Clindamycin was used as prophylaxis in both pre- and intervention periods. Also, a subset of patients in both the pre- and intervention group received topical gentamicin irrigation, based on the individual neurosurgeon’s decision. No other change in the preoperative preparations, surgical procedure(s), operating rooms or postoperative care was made during the study period. The study was approved by the Stockholm regional ethical review board.

### Study population

All patients undergoing elective tumour surgery with a craniotomy procedure at Karolinska University Hospital (Stockholm, Sweden) during the periods May 2012–January 2013 and May 2013–January 2014 (pre-intervention period, a total of 18 months) and May 2014–January 2015 (intervention period, 9 months) were identified. The intervention period started with the change of antibiotic prophylaxis 1st of May 2014 and was terminated the 31st of January 2015, since a possibly interfering study on the ventilation system of the operating rooms started at the department on 1st of February 2015. While it has been shown that SSI has seasonal variations [3, 26], it was decided to include pre-intervention patients from the same period of the year, May–January. Data from May to January during two consecutive years were collected in the pre-intervention group to reach enough number of patients. Karolinska University Hospital serves a population of 2.1 million habitants. Patients with intracranial tumours in the Stockholm and Gotland region are exclusively treated at Karolinska University Hospital ensuring a population-based material.

Exclusion criteria were surgery performed on an emergency basis and/or previous surgery < 12 months from the index operation. A total of 375 patients were included in the pre-intervention group and 205 patients were included in the intervention group.

To rule out a possible clinical trial effect (“Hawthorne effect”) [13], a control group from Department of Neurosurgery at Sahlgrenska University Hospital (Gothenburg, Sweden) was reviewed. At Sahlgrenska, the opposite change in antibiotic prophylaxis policy (i.e., a change from Cefuroxime to Cloxacillin) was made in 2010, as the result of a new clinical guideline on CNS infections published by the Swedish society of infectious diseases [9]. At the time, limited use of second generation cephalosporins to reduce the risk of antibiotic resistance was recommended by the Swedish Reference group for antibiotic issues (RAF) [17]. Operation logs for all patients treated with craniotomy for intracranial tumours during 2005–2009 and 2011–2014, respectively, were reviewed for the prevalence of reoperations due to SSI.

### Data collection

Clinical and demographic data, including known risk factors for SSI, were retrieved by retrospective review of digital patient records. Data collection continued at least 12 months postoperatively.

### Endpoints

All endpoints were defined a priori. The primary endpoint was a comparison of SSI rate between the pre-intervention (Cloxacillin prophylaxis) group and the intervention (Cefuroxime prophylaxis) group. SSI was defined according to the Center for Disease Control’s *Guideline for prevention of surgical site infection*, 1999 [21]. The craniotomy procedure implies insertion of implants to reattach the bone flap; hence, data were registered at 3 and 12 months according to CDC’s 1999 guidelines. The Neurosurgical Department at Karolinska University Hospital is the only neurosurgical unit in the study region; all major complications after neurosurgery are reported or referred to Karolinska. To still rule out the possibility of missing data on SSI incidence, patient charts from regional hospitals and general practitioners in the region were also reviewed.

Secondary endpoints included the reoperation rate, the amount of antibiotics used (i.e. SSI treatment, excluding prophylactic antibiotic) and the number of visits in the outpatient clinic due to SSI.

### Statistical analysis

Primary endpoints were analysed both according to the actual treatment provided (per protocol) as well as according to treatment policy (intention to treat). Statistical significance level was set to *p* < 0.05.

Comparisons of dichotomous data (including primary endpoint) were analysed with Pearson Chi-square test or Fisher’s exact test (when expected frequencies < 5). Distributions of continuous variables were analysed with Q-Q plots. For continuous data, comparisons of groups were analysed with independent sample *t* test if normally distributed or with Mann-Whitney *U* test if skewed. Log rank test was used to analyse Kaplan-Meier curves. Logistic regression was used for control of covariates. Statistical analyses were performed using SPSS Statistics for Windows, Version 18.0 (Chicago: SPSS Inc.)

## Results

A comparison of demographic and clinical characteristics for the pre- and intervention groups is presented in Table 1. The groups were comparable except that duration of surgery was longer and the use of intraoperative Gentamicin solution was more frequent prior to the intervention, while the Charlson comorbidity index (CCI > 2) was higher in the intervention group.

**Table 1:** Demographic and clinical characteristics of groups

Characteristic	Pre-intervention group (*n* = 375)	Intervention group (*n* = 205)	*P* value
Age at surgery, mean (SD)	54.2 (14.8)	56.0 (15.5)	0.17
Sex, female *n* (%)	199 (53.1)	116 (56.6)	0.42
Diagnosis/type of surgery			0.72
Benign tumour, *n* (%)	163 (43.5)	86 (42.0)	
Malignant tumour, *n* (%)	212 (56.5)	119 (58.0)	
AB prophylaxis			
Cloxacillin, *n* (%)	326 (86.9)	5 (2.4)	< 0.01
Cefuroxim, *n* (%)	17 (4.5)	189 (92.2)	< 0.01
Clindamycin, *n* (%)	32 (8.5)	11 (5.4)	0.19
Gentamicin intraoperatively, *n* (%)	112 (29.9)	33 (16.1)	< 0.01
Operation time, median (IQR)	201 (142–306)	182 (122–269)	0.05
Body mass index, median (IQR)	25 (23–28)	26 (22–29)	0.59
S-Albumin, mean (SD)	37 (4.5)	36.5 (4.5)	0.18
Smoking, *n* (%)	45 (12.0)	24 (11.7)	0.08
Length of stay, mean days (SD)	9.6 (6.6)	9.3 (6.0)	0.59
Reoperation (index), *n* (%)	52 (13.9)	23 (11.2)	0.36
Charlson comorbidity index > 1, *n* (%)	188 (50.1)	124 (60.5)	0.02
ASA class > 2, *n* (%)	343 (91.5)	189 (93.2)	0.47
Preoperative steroids, *n* (%)	357 (95.2)	191 (93.2)	0.25

### SSI

**Fig. 1:**
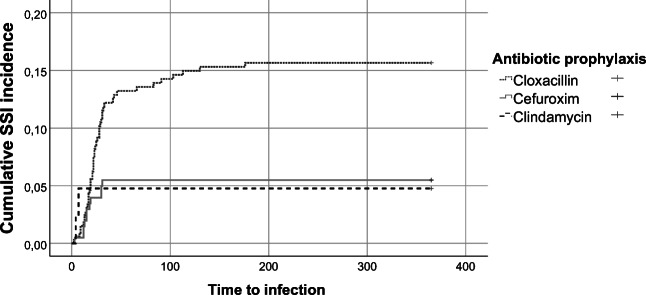
Kaplan-Meier curve of SSI following elective craniotomy for brain tumour surgery pre-intervention and intervention analysed by intention to treat

**Fig. 2:**
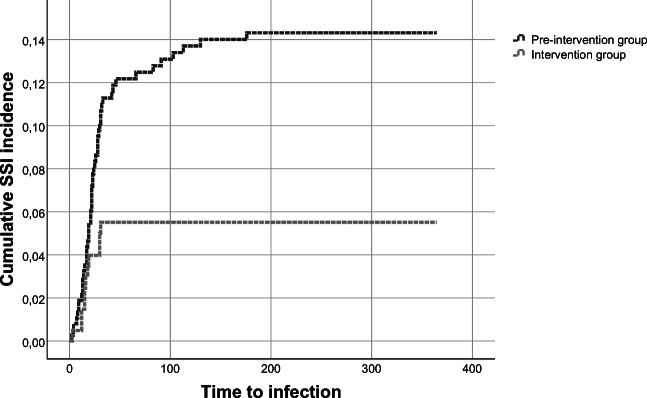
Kaplan-Meier curve of SSI following elective craniotomy for brain tumour surgery per protocol

The pre-intervention group had a significant higher incidence of SSI, with 50/375 patients (13.3%) compared to 11/205 (5.4%) in the post-intervention group (*p* < 0.01). When analysed per protocol, the risk of SSI in the group that received preoperative Cloxacillin was 14.5% (48/331). The corresponding risk in the group with Cefuroxime prophylaxis was 5.3% (11/206) (*p* < 0.01). The primary end-point is presented in Table 2 and in Kaplan-Meier curves (Figs. 1 and 2) (intention to treat and per protocol).

**Table 2:** Surgical site infections, infection depth, bacteria type and treatments by pre-intervention and intervention group

Variable	Pre-intervention group (*n* = 375)	Intervention group (*n* = 205)	*P* value
SSI, intention to treat, *n* (%)	50 (13.3)	11 (5.4)	< 0.01
SSI per protocol, *n* (%)	48 (14.5)	11 (5.3)	< 0.01
Reoperation due to SSI *n*, (%)	31 (8.3)	6 (2.9)	0.02
Type of reoperation, *n* (%)			
Skin wound revision	4 (1.1)	2 (1.0)	0.99
Bone flap removal	7 (1.9)	1 (0.5)	0.27
Intradural operation	20 (5.3)	4 (2.0)	0.05
Depth of SSI, *n* (%)			
Superficial	20 (5.3)	4 (2.0)	0.05
Deep	3 (0.8)	1 (0.5)	0.99
Organ/space	27 (7.2)	6 (2.9)	0.04
Use of additional iv antibiotics, days/100 operations	160	74	0.07
Use of additional po antibiotics, days/100 operations	445	114	< 0.01
Total use of additional antibiotics (IV + PO), days/100 operations	605	188	0.03
Additional outpatient visits, visits/100 operations	33	10	< 0.01
Bacteria type, *n* (%)^a^			
*Staphylococcus aureus*	10 (2.7)	3 (1.5)	0.56
Coagulase-negative staphylococci	3 (0.8)	2 (1.0)	0.99
Proprionebacterium acnes	22 (5.9)	4 (2.0)	0.04
Other species	8 (1.8)	2 (1.0)	0.51
Negative culture	12 (3.2)	1 (0.5)	0.43

^a^A total of 6 patients had cultures with more than bacteria type identified

### Control group

In the control group, the rate of reoperation due to SSI was reviewed during the years 2005–2009, when Cefuroxime was used as prophylaxis and compared to the years 2011–2014 when Cloxacillin was used as prophylaxis. The reoperation rate due to SSI was significantly lower when cefuroxime was routinely used as antibiotic prophylaxis during the years 2005–2009 than during 2011–2014 when antibiotic prophylaxis was changed to Cloxacillin; 1.8% (27/1529) vs 3.1% (43/1378), respectively (*p* = 0.02).

### Secondary outcomes

The secondary outcomes and characteristics of the patients with SSI are summarized in Table 2. The number of reoperations due to SSI were significantly lower post intervention, 7/205 (3.4%) versus 31/375 (8.3%) *P* = 0.02. Also, a significant reduction of the total use of antibiotics (i.e. SSI treatment excluding pre-operative prophylaxis) was observed, 605 vs 188 days with antibiotic treatment per 100 operations, *p* = 0.03. The number of visits in the outpatient clinic due to SSI were also significantly reduced from 33 in the pre-intervention group in comparison to 10 in the intervention group, *p* = 0.001.

### Bacterial cultures

Significantly less infections caused by *Propionibacterium acnes* could be seen after the intervention. Also, in the pre-intervention group, more cultures were negative or missing (12 vs 1), mostly due to a greater number of superficial incisional infections where antibiotics had been started empirically before cultures were secured.

## Discussion

In this quasi-experimental study, a significant reduction of SSIs and numbers of reoperations due to SSI was seen when the pre-operative antibiotic prophylaxis regimen was changed from Cloxacillin to Cefuroxime. Also, outpatient visits due to SSI and total use of antibiotics were reduced after the regimen changed. These results were supported by increased numbers of reoperations due to SSI in a comparable control group where the opposite change, from Cefuroxime to Cloxacillin, was made.

Currently, all major national and international neurosurgical guidelines recommend antibiotic prophylaxis for neurosurgical procedures [20]. The selection of prophylactic agent is usually based on the surgical procedure and what pathogens are most likely to cause infection for the wound type. Additionally, factors such as the agent’s bactericidal activity, safety, hospital/regional resistance patterns and cost are weighed in. In neurosurgery, the pharmacokinetic profile of the agent, especially its ability to pass the blood-brain barrier and the resulting concentration in cerebrospinal fluid, is crucial. The ideal prophylactic antimicrobial agent has the narrowest spectrum of activity possible, while still targeting the most common organisms [31]. Today, most guidelines recommend a cephalosporin as antibiotic prophylaxis in neurosurgical procedures, with Cefazolin (a third-generation cephalosporin) being most frequent [20].

Cloxacillin is a penicillinase-resistant penicillin proven to be an effective prophylactic antimicrobial agent in neurosurgery [32–34]. With a relatively narrow spectrum, Cloxacillin has a favourable profile regarding side effects and the risk of developing resistant organisms. It targets *Staphylococcus* and *Streptococcal* species, including the most common infectious agent in neurosurgical SSI: *S. aureus*. However, due to increasing bacterial resistance, the use of Cloxacillin as prophylaxis in neurosurgical procedures has decreased [20]. The use of Cloxacillin as prophylaxis in our department at Karolinska University Hospital was questioned as increasing infection rates were noted in 2013 and early 2014. It was decided to update the prophylactic antibiotic regimen and the choice fell on Cefuroxime.

The second-generation cephalosporin Cefuroxime has several theoretical advantages over Cloxacillin as prophylaxis in neurosurgery; it has a longer half-life (1.3 h vs 0.5 h) and thus prolonged serum concentrations [18], it has a protein binding grade of 33–50% compared to Cloxacillin’s 95%, which yields a higher unbound plasma concentration that can pass the blood-brain barrier [24], and it is more hydrophobic, facilitating penetration to the CNS [27]. Cerebrospinal fluid concentrations of cefuroxime correlate with the degree of inflammation and are considerable higher in patients with meningitis [14, 18]. However, even without meningeal inflammation, the concentration reaches levels enough to inhibit bacteria to a sufficient degree [19]. Compared to Cloxacillin, Cefuroxime also has a broader antimicrobial spectrum, not only covering gram positives as *Staphylococcus* and *Streptococcus* species, but also gram negative bacilli commonly causing SSI [18].

Possible negative effects of using Cefuroxime as prophylaxis compared to Cloxacillin are related to the former’s broader antimicrobial spectrum, with a greater impact on intestinal flora and a higher risk of emergence of resistant microorganisms [2]. However, in the event of an SSI, long-term treatment with antibiotics is needed, often with combinations of drugs with even broader spectra such as Vancomycin and Meropenem. In our study, the total use of antibiotics was significantly reduced in the intervention group receiving Cefuroxime, advocating the choice of a slightly broader spectrum antibiotic as prophylaxis. Also, Cefuroxime has proven a well-tolerated and safe drug in terms of side effects.

The significantly lower SSI rate in our intervention group was to a considerable extent attributed to the reduction of *P. acnes* infections (Table 2). As a possible explanation, the above-mentioned properties of Cefuroxime, such as prolonged serum concentrations due to Cefuroxime’s longer half-life, may give a better protection against contaminating skin bacteria during surgery and in the immediate postoperative period compared to Cloxacillin. The amount of negative or missing cultures was also considerably lower in the post-intervention group. These patients generally had superficial SSIs where antibiotic treatment had been initiated without securing cultures, or cultures were taken with ongoing antibiotic therapy. The lack of known infectious agent in almost one third of the SSIs in the pre-intervention group makes it harder to interpret the results; nevertheless, all reported infections fulfilled the criteria of a SSI event as defined by CDC [21].

### Limitations

The retrospective data collection in this study, with historical controls, makes it prone to Hawthorne effects. However, the validity of the results is strengthened by the increase of reoperations due to SSI in a control group at the Sahlgrenska University Hospital, where the opposite change from Cefuroxime to Cloxacillin was made.

The SSI incidence and reoperation rate reported in this study’s both treatment arms are in the higher end of SSI rates reported in previous studies [1, 5, 25, 30]. This could to some extent be explained by the study design. Our study was conducted in a defined geographic catchment area, served by a single neurosurgical centre in a region that employs a common electronical medical records system. It is likely that all post-operative infections were recorded, with no or negligible under-reporting. This is also supported by the fact that neurosurgical studies with similar population-based designs have shown higher complication rates than commonly reported [6, 15, 29]. Nevertheless, the rates could be considered extremes, and one could expect part of the results to be attributed to a regression to the mean phenomenon. In the Sahlgrenska control group, however, reoperation rates due to SSI were modest, and still a significant effect of the changed antibiotic regimen could be seen.

No other changes than those of the antibiotic regimen were made in the treatment of intracranial tumours at our department during the study period. Still, there are possible confounding factors not controlled for, such as the impact of individual surgeons and variations in surgical technique. Noteworthy, the median operation time—a known risk factor for SSI—was significantly longer in the pre-intervention group. However, other factors favoured the pre-intervention group, such as a significant higher use of topical gentamicin irrigation [36] in the pre-intervention group and significantly higher comorbidity in the intervention group, and thus the impact on the results from the recorded demographic and clinical factors should be low.

This study includes patients operated for intracranial tumours only. Tumour surgery is in general non-emergent, clean and has standardized pre- and postoperative routines. By studying this subgroup, possible confounding factors such as preoperative contamination or prolonged postoperative drain treatment, can be kept to a minimum. Even though changed antibiotic prophylaxis from Cloxacillin to Cefuroxime is likely to have a similar effect on other types of intracranial surgery, it is not investigated in this study and caution is advised when interpreting the results.

Local variations of bacterial resistance patterns affect the generalizability of the results in this study. Regional and even hospital bacterial resistance patterns must be taken in consideration when choosing the type of antibiotic prophylaxis.

## Conclusion

Surgical site infections and reoperations due to SSI were significantly reduced when cefuroxime was used compared to cloxacillin. Also, the total amount antibiotics used and the number of visits in the outpatient clinic was significantly lowered. Cefuroxime seems to be a more effective antibiotic prophylaxis than Cloxacillin in intracranial tumour surgery by craniotomy.
